# Rapid, Quantitative, High-Sensitive Detection of *Escherichia coli* O157:H7 by Gold-Shell Silica-Core Nanospheres-Based Surface-Enhanced Raman Scattering Lateral Flow Immunoassay

**DOI:** 10.3389/fmicb.2020.596005

**Published:** 2020-11-06

**Authors:** Luoluo Shi, Ling Xu, Rui Xiao, Zihui Zhou, Chongwen Wang, Shengqi Wang, Bing Gu

**Affiliations:** ^1^Medical Technology Institute of Xuzhou Medical University, Xuzhou, China; ^2^Beijing Institute of Radiation Medicine, Beijing, China; ^3^Department of Laboratory Medicine, Affiliated Hospital of Xuzhou Medical University, Xuzhou, China; ^4^College of Life Sciences, Anhui Agricultural University, Hefei, China

**Keywords:** SERS, lateral flow immunoassay, *E. coli* O157:H7, SiO_2_/Au, quantitative detection

## Abstract

*Escherichia coli* O157:H7 is regarded as one of the most harmful pathogenic microorganisms related to foodborne diseases. This paper proposes a rapid-detection biosensor for the sensitive and quantitative analysis of *E. coli* O157:H7 in biological samples by surface-enhanced Raman scattering (SERS)-based lateral flow immunoassay (LFIA). A novel gold-shell silica-core (SiO_2_/Au) nanosphere (NP) with monodispersity, good stability, and excellent SERS activity was utilized to prepare high-performance tags for the SERS-based LFIA system. The SiO_2_/Au SERS tags, which were modified with two layers of Raman reporter molecules and monoclonal antibodies, effectively bind with *E. coli* O157:H7 and form sandwich immune complexes on the test lines. *E. coli* O157:H7 was quantitatively detected easily by detecting the Raman intensity of the test lines. Under optimal conditions, the limit of detection (LOD) of the SiO_2_/Au-based SERS-LIFA strips for the target bacteria was 50 cells/mL in PBS solution, indicating these strips are 2,000 times more sensitive than colloidal Au-based LFIA strips. Moreover, the proposed assay demonstrated high applicability in *E. coli* O157:H7 detection in biological samples, including tap water, milk, human urine, lettuce extract and beef, with a low LOD of 100 cells/mL. Results indicate that the proposed SERS-based LFIA strip is applicable for the sensitive and quantitative determination of *E. coli* O157:H7.

## Introduction

As one of the major foodborne pathogens that cause food poisoning and serious illnesses worldwide, *Escherichia coli* O157:H7 is a significant threat to public health (Suaifan et al., 2017; Hassan et al., 2019). *E. coli* O157:H7 can exist in sewage-tainted water, contaminative milk, and meat products. It can cause bloody diarrhea, urinary tract infection, and hemolytic uremic syndrome, and its infectious dose is as low as 10 cells (Song et al., 2016; Shin et al., 2018). Rapid and sensitive methods that could timely diagnose this pathogen are the key to reduce the spread of infection and guarantee food safety at the source. The traditional microbiological culture method is considered as the standard method for bacterial detection (Hu et al., 2016; Li et al., 2019). However, it is labor intensive and time consuming, usually needing more than 24 h of culture and analysis. Some modern methods, such as enzyme-linked immunosorbent assay (ELISA), polymerase chain reaction (PCR), bioluminescence signal, DNA sequencing-based test, and mass spectrometry (MS), have been recently developed and successfully applied in laboratory testing (Jia et al., 2015; Guo et al., 2016; Sauget et al., 2017; Liu et al., 2018). These methods are fairly rapid and accurate but have some disadvantages, such as tedious procedures, sample pretreating, and strict laboratory conditions (Law et al., 2014). Thus, a sensitive and convenient point-of-care testing (POCT) must be developed for *E. coli* O157:H7 detection.

Lateral flow immunoassay (LFIA), which possesses the advantages of short testing time, low cost, user-friendly format, and portability in diverse applications, has been considered one of the most promising POCT methods (Wang et al., 2018b, 2019a; Zhang et al., 2019; Mahmoudi et al., 2020). However, the inherent defects of conventional LFIA are poor quantitative ability and limited detection sensitivity, which both depend on colorimetric analysis. Increasing attention has been paid to surface-enhanced Raman Scattering (SERS)-based LFIA technology, which combines the high sensitivity and quantitative analysis of SERS technology and the rapidity and convenience of LIFA (Hwang et al., 2016; Lee et al., 2019; Wang et al., 2019b). SERS-based LFIA uses Raman reporter-labeled SERS nanomaterials instead of colloidal Au as the signal tags, thereby providing strong and stable SERS signals (Jia et al., 2018; Khlebtsov et al., 2018). SERS-based LFIA has been successfully applied in the sensitive and quantitative analysis of various targets, such as tumor biomarkers, heart disease biomarkers, toxins, DNA markers, and viruses (Maneeprakorn et al., 2016; Wang X. et al., 2017; Zhang et al., 2018a,b; Lu et al., 2020). However, some difficulties must be overcome in detecting *E. coli* O157:H7 via SERS-based LFIA. First, the pores of the NC membrane of LFIA strips are easily blocked by the size of *E. coli* (generally above 1 μm), resulting in strong non-specific signal or false-positive outcome on the test line. Second, food or clinical samples contain abundant matrix interferences, which may affect the flow of tags on the strips and decrease the sensitivity of the SERS-based LFIA system (Cho et al., 2015; Wang et al., 2020). Thus, high-performance SERS tags with excellent stability in complex samples and good dispersibility on the strip are needed to solve these problems.

In this study, a new type of dual Raman molecules loaded with Au-shell SiO_2_-core nanospheres (SiO_2_/Au NPs) was synthesized and introduced into the LFIA system as high-performance SERS labels for *E. coli* detection. The SiO_2_/Au tags possessed great dispersity and stability in complex samples and provided stable and intense SERS signal on the test zone of the LFIA system, which could meet the actual requirements for bacterial detection using SERS-based LFIA. By recording the SERS signal of the test line of the proposed SERS-based LFIA, *E. coli* O157:H7 could be quantitatively detected within 15 min. The sensitivity of the SiO_2_/Au-based SERS-LFIA strips for *E. coli* O157:H7 detection was 50 cells/mL in PBS solution, which was 2000 times higher than that of colloidal Au-based LFIA strips. Furthermore, the proposed SERS-based LFIA strips were successfully applied to tap water, milk, human urine, lettuce extract and beef samples. A low limit of detection (LOD) of 100 cells/mL was achieved because of the prominent stability of the synthesized SiO_2_/Au tags, indicating their good practicability in food security and clinical diagnosis.

## Experimental

### Chemicals and Materials

Chlorauric acid tetrahydrate (HAuCl_4_⋅4H_2_O), hydroxylamine hydrochloride, sodium borohydride, ammonia solution (28%, w/w) and trisodium citrate were purchased from Sinopharm Chemical Reagent Co. (Shanghai, China)^1^. Tetraethoxysylane (TEOS), polyethyleneimine (PEI), 5,5′-dithiobis-(2-nitrobenzoic acid) (DTNB), polyvinylpyrrolidone (PVP, 40 K), N-(3-dimethylaminopropyl)-N′-ethylcarbodiimide hydrochloride (EDC), N-hydroxysuccinimide (NHS), MES monohydrate, bovine serum albumin (BSA), phosphate buffer (PB, 0.1 M) and Tween-20 were purchased from Sigma-Aldrich (Shanghai, China)^2^. Fetal bovine serum (FBS) and phosphate buffer saline (PBS, 10 mM) were obtained from Thermo Fisher (Shanghai, China)^3^. Goat anti-mouse IgG antibody and mouse monoclonal antibody to *E. coli* O157:H7 were bought from ACTHTEAM (Changzhou, China)^4^. Sample pad, absorbent pad, conjugate pad, PVC bottom plate and 50 nm colloidal Au NPs were bought from Jieyi Biotechnology Co. (Shanghai, China)^5^. Nitrocellulose membrane were purchased from Sartorius (Shanghai, China)^6^. *E. coli* O157:H7 (ATCC 35150), *enteroaggregative E. coli* (*EAEC*, BNCC340663), *enteroinvasive E. coli* (*EIEC*, BNCC340975), *enteropathogenic E. coli* (*EPEC*, BNCC186732), *enterotoxigenic E. coli* (*ETEC*, BNCC186736), and *E. coli* BL21 (BNCC353806) was obtained from BeiNa Biotechnology Co., Ltd. (Beijing, China)^7^. The clinical isolates of *Staphylococcus aureus* (*S. aureus*), *Salmonella enterobacter* (*S. enterobacter*), *Shigella flexneri* (*S. flexneri*), *Listeria monocytogenes* (*L. monocytogenes*), *Vibrio cholerae* (*V. cholerae*), and *Enterococcus faecalis* (*E. faecalis*) were obtained from The Affiliated Hospital of Xuzhou Medical University.

### Instruments

Transmission electron microscope (TEM) images were obtained with a Hitachi H-7650 TEM operated at 150 kV. The high-resolution transmission electron microscope (HRTEM) images were acquired via a Philips Tecnai G2 F20 microscope at an acceleration voltage of 200 kV. Scanning electron microscope (SEM) images were obtained from a JTOL JSM-7001F microscope operated at 10 kV. UV-Vis spectra were obtained with a Shimadzu 2600 spectrometer. Zeta potentials were measured by a Malvern Nano-ZS90 Zetasizer. Raman spectra was recorded by a B&W Tek portable Raman spectrometer (i-Raman Plus BWS465-785H). All the samples were excited with a 785 nm laser, which laser power and acquisition time at each spot was 10 mW and 10 s, respectively.

### Synthesis of Dual DTNB-Modified SiO_2_/Au NPs

**SCHEME 1:**
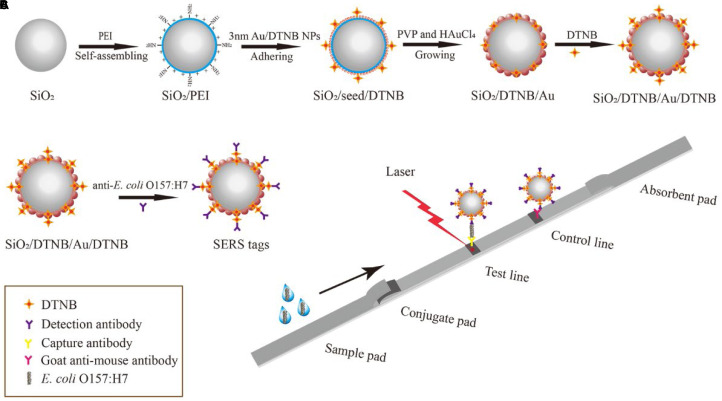
Schematic of the preparation of dual DTNB-modified SiO_2_/Au NPs **(A)**, SiO_2_/Au SERS tags **(B)**, and SERS-based LFIA strip **(C)**.

The fabrication principle of dual DTNB-modified SiO_2_/Au NPs is shown in Scheme 1A. The SiO_2_/DTNB/Au/DTNB NPs were synthesized using our previously reported seed-mediated growth method with some modifications. First, SiO_2_ NPs were prepared via a modified Stöber method (Wang C. et al., 2017). Subsequently, 0.5 mL of SiO_2_ NPs (7 mg/mL) were added to 100 mL of deionized water and ultrasonically treated for 10 min. Then, 10 mL of PEI solution (0.5 mg/mL) was added into the mixture and ultrasonically treated for 40 min to form SiO_2_/PEI NPs. The obtained SiO_2_/PEI NPs were centrifuged and washed two times using deionized water to remove excess PEI. Afterward, 3–5 nm Au NPs were prepared in accordance with the method of Fang et al. (2010) and modified with DTNB (30 μM). The obtained SiO_2_/PEI NPs were added into 150 mL of 3–5 nm Au/DTNB NPs and ultrasonically treated for 30 min, forming SiO_2_/seed/DTNB microspheres. The obtained SiO_2_/seed/DTNB NPs were centrifuged and washed using deionized water to eliminate the superfluous 3–5 nm Au/DTNB NPs and kept in 5 mL of anhydrous ethyl alcohol.

The SiO_2_/DTNB/Au NPs were synthesized with an ultrasonic cleaner (800 W power) within 5 min. In brief, 2.5 mL of the obtained SiO_2_/seed/DTNB NPs were added into 100 mL of aqueous solution containing hydroxylamine hydrochloride (0.5 mg/mL) and PVP (2 mg/mL). The mixture was ultrasonically treated for 5 min and 206 μL of HAuCl_4_ (1 wt.%) was added and ultrasonically treated for another 5 min. Then, the obtained SiO_2_/DTNB/Au NPs were centrifuged and washed two times using deionized water to remove the superfluous PVP and kept in 5 mL of anhydrous alcohol. Finally, the obtained SiO_2_/DTNB/Au NPs were ultrasonically treated with DTNB ethanol solution (50 μL, 10 mM) for 1 h to form dual DTNB-modified SiO_2_/Au NPs.

### Preparation of Dual DTNB-Modified SiO_2_/Au SERS Tags

Dual DTNB-modified SiO_2_/Au NP SERS tags were prepared via EDC/sulfo-NHS, where the DTNB-COOH group is combined with the amide group of the antibody (Scheme 1B). In brief, 1 mL of the dual DTNB-modified SiO_2_/Au NPs were centrifuged and washed using deionized water to eliminate the superfluous DTNB and alcohol. Afterward, the pellet was resuspended in 1 mL of MES buffer (0.1 M, pH 5.5). Freshly prepared EDC (10 mM, 100 μL) and NHS (100 mM, 20 μL) were added into the mixture and ultrasonically treated for 15 min. Subsequently, the mixture was centrifuged and resuspended in 1 mL of 0.05% PBST solution (PBS containing 0.05% Tween-20, v/v). *E. coli* O157:H7 detection antibody (20 μg) was added into the mixture and incubated for 2 h. Then, 100 μL of 10% BSA (w/v) was added to block the unreacted carboxyl sites of SERS labels for an additional 1 h at 37°C. The functionalized SERS labels were pelleted by centrifugation and resuspended in 500 μL of a solution containing Tris–HCl (50 mM, pH 8.0), 0.5% Tween-20 (v/v), 1% BSA (w/v), 10% sucrose (w/v), and 0.1% PVP (w/v). The SERS tags were evenly pipetted onto the glass fiber paper and dried at 37°C to form conjugate pads.

### Preparation of Dual DTNB-Modified SiO_2_/Au-Based SERS-LFIA Strips

The SERS-based LFIA strip was composed of four sections: sample pad, absorbent pad, conjugate pad with SERS tags, and NC membrane (test and control lines). *E. coli* O157:H7 capture antibody (0.4 mg/mL) and goat anti-mouse IgG antibody (1 mg/mL) were drawn onto the NC membrane pad separately and dried at 37°C for 1 h. Then, the four sections were assembled onto a PVC bottom plate in sequence. The integrated membrane was cut into 3 mm-wide strips with a paper cutter and stored in a dry container until use.

### Preparation of Bacterial Sample

The standard strains were cultivated in 5 mL of Luria–Bertani broth at 37°C for 5 h. The *E. coli* O157:H7 concentration was verified via plate count method. In brief, the original bacterial solution was diluted 10^5^, 10^6^, and 10^7^ times into 1 mL of sterile water, and 200 μL of which was added onto blood agar plates and cultured for 12 h, as shown in Supplementary Figure S1. The original *E. coli* O157:H7 concentration was 1.145 × 10^8^ cells/mL.

### *E. coli* O157:H7 Detection Using Dual DTNB-Modified SiO_2_/Au-Based SERS-LFIA Strips

The detection process of the LFIA strips is shown in Scheme 1C. *E. coli* O157:H7 was diluted in running buffer (PBS containing 20% FBS and 0.5% Tween-20, v/v), with concentrations ranging from 50 cells/mL to 10^7^ cells/mL, as positive samples. The running buffer without *E. coli* O157:H7 was used as the blank control. Under the capillary effect, the sample solution (100 μL) moved toward the absorbent pad when dropped onto the sample pad. The test process was completed within 15 min. The SERS signals of the test lines were tested with a portable Raman spectrometer, with integration time and laser power of 10 s and 10 mW, respectively.

### *E. coli* O157:H7 Detection in Biological Samples

The preparation of lettuce extract and beef samples was done as described by Li et al. (2020). *E. coli* O157:H7 was spiked into tap water, milk, human urine, the lettuce extract and beef samples, with concentrations ranging from 50 cells/mL to 10^7^ cells/mL. The detection process was similar to that of *E. coli* in running buffer but needed an additional diluent (FBS containing 2.5% Tween-20, v/v). In brief, 80 μL of the sample was adequately mixed with 20 μL diluent. The mixture was then pipetted onto the sample pad of the SERS-based LIFA strips. Twelve min later, the test lines were tested with a portable Raman spectrometer.

## Results and Discussion

### Characterization of Dual DTNB-Modified SiO_2_/Au SERS Tags

**FIGURE 1:**
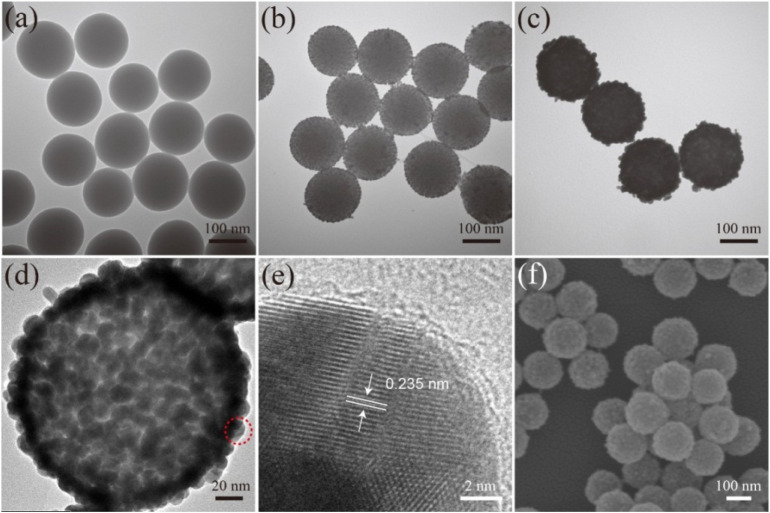
Characterization of synthesis SiO_2_/Au NPs. TEM images of SiO_2_ **(a)**, SiO_2_/PEI-seed **(b)**, and SiO_2_/Au **(c)**. HRTEM images **(d,e)** and SEM image **(f)** of SiO_2_/Au NPs.

The products synthesized at different stages were analyzed via TEM and SEM to characterize their structure and morphology. The monodispersed SiO_2_ NPs (Figure 1A) prepared using the modified Stöber method had a uniform diameter size of approximately 150 nm. After the SiO_2_ NPs were coated with PEI layer, the 3–5 nm Au/DTNB NPs densely adhered on the surface of the SiO_2_/PEI NPs (Figure 1B). These small Au NPs acted as nucleation sites for Au shell synthesis. Figure 1C displays the TEM image of the obtained SiO_2_/DTNB/Au NPs, whose size increased from 150 nm to approximately 180 nm. The Au shell was approximately 15 nm thick. Figures 1B,C illustrate the strong difference between the SiO_2_/seed NPs and the SiO_2_/Au NPs. Figures 1D,F show the HRTEM and SEM images of the SiO_2_/DTNB/Au NPs, respectively, displaying the rough surface of the SiO_2_/DTNB/Au NPs. The dense Au/DTNB NPs obviously increased in size and formed a rough Au shell on the surface of the SiO_2_ particles, thereby ensuring high SERS performance. Figure 1E illustrates the HRTEM image of a part of Au shell. The distance of the crystal planes was 0.235 nm, corresponding to the Au(111) plane (Switzer et al., 2016; Wang et al., 2016).

**FIGURE 2:**
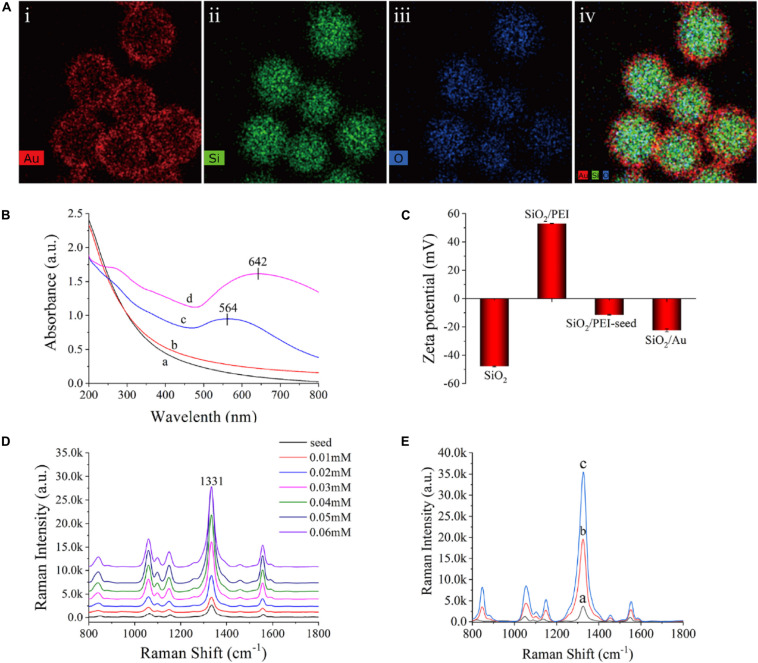
**(A)** Element mapping of SiO_2_/Au NPs (i–iv). **(B)** UV-vis spectra of SiO_2_ (curve a), SiO_2_/PEI (curve b), SiO_2_/PEI-seed (curve c), and SiO_2_/Au (curve d). **(C)** Zeta potential of SiO_2_, SiO_2_/PEI, SiO_2_/PEI-seed, and SiO_2_/Au. **(D)** Raman intensity of SiO_2_/seed/DTNB/Au NPs with different concentrations of HAuCl_4_: 0.01, 0.02, 0.03, 0.04, 0.05, and 0.06 mM. **(E)** Raman spectra of SiO_2_/PEI-seed/DTNB (curve a), SiO_2_/Au/DTNB (curve b), and SiO_2_/DTNB/Au/DTNB (curve c).

Figure 2A shows the element mapping of the SiO_2_/Au NPs. Si (green) and O (blue) were surrounded by dense Au (red), indicating that the SiO_2_ microspheres were successfully coated with an Au shell. The synthesized products at different stages were measured in deionized water with a UV-vis spectrometer, as shown in Figure 2B. The SiO_2_ NPs and SiO_2_/PEI NPs did not show distinct absorption peaks (curves a and b), whereas the SiO_2_/PEI-seed had a distinct absorption peak at approximately 564 nm (curve c). This finding suggested that several small Au/DTNB seeds were successfully absorbed on the surface of SiO_2_ NPs. After an Au shell was formed, the absorption peak in the UV-vis spectra shifted to 642 nm, and its intensity increased simultaneously (curve d). As shown in Figure 2C, the synthesized products at different stages were tested in deionized water with a zeta potential analyzer. The zeta potential of the SiO_2_ NPs increased from −47.8 to 53.0 mV when the PEI was modified on the silicon spheres. This change confirmed that the surface of the SiO_2_ NPs successfully absorbed a layer of positive-charged PEI. The zeta potential became negative again when small Au/DTNB NPs were attached to the SiO_2_/PEI particles (−11.5 mV). The negative potential increased further when an Au shell was complete formed (−22.4 mV).

The method for forming Au shell is basically seed-mediated growth. The thickness of the Au shell could be easily controlled by adjusting the concentration of HAuCl_4_ when the amount of SiO_2_/PEI-seed NPs remained constant. Supplementary Figure S2 exhibits the representative TEM images of SiO_2_/seed/DTNB/Au NPs, which were fabricated with different concentrations of HAuCl_4_ when other conditions were kept invariable. The 3–5 nm Au/DTNB NPs attached to the SiO_2_ spheres gradually increased in size and finally connected with one another to completely form an Au shell with different thicknesses when the concentrations of HAuCl_4_ increased from 0.01 to 0.06 mM. The Raman intensity of the SiO_2_/seed/DTNB/Au NPs was tested to investigate the influence of Au shell thickness on SERS performance. Figure 2D exhibits the SERS activity of the SiO_2_/seed/DTNB/Au NPs with different Au shell thicknesses. Compared with the poor SERS performance of SiO_2_/seed NPs, the SERS enhancement ability of the SiO_2_/seed/DTNB/Au NPs gradually increased along with the thickening of the Au shell. However, the SERS ability of SiO_2_/seed/DTNB/Au NPs decreased when the Au shell overgrew, decreasing the hot spots and nanogaps on the surface of the SiO_2_/Au NPs. Therefore, the SiO_2_/Au NPs prepared with 0.05 mM HAuCl_4_ was chosen as the ideal material for SERS application because its Au shell was almost complete and the SERS performance was optimal. Notably, we selected DTNB as the Raman reporter molecule to build SiO_2_/Au SERS tags, because DTNB can be easily modified on the Au shell through Au–S covalent bonding and provide a large Raman cross section (Song et al., 2018; Zhang C. et al., 2018; Pang et al., 2019).

Figure 2E shows the SERS activity of the SiO_2_/PEI-seed, SiO_2_/Au, and SiO_2_/DTNB/Au NPs. The SERS activity of the SiO_2_/PEI-seed NPs was fairly weak but increased when an Au shell was completely formed. The SERS activity further increased as DTNB was modified inside the Au shell (Rong et al., 2018). This phenomenon could be due to two reasons. First, a mass of hot spots existed in the rough Au shell. Second, increased amounts of DTNB molecules were embedded inside the Au shell. However, once the amount of DTNB molecules on the surface of the SiO_2_/PEI-seeds was in excess, an Au shell could not be completely fabricated outside the SiO_2_ NPs, as shown in Supplementary Figure S3A. Compared with common colloidal Au NPs, the SiO_2_/PEI-seed/DTNB/Au NPs could work in high ion solution and be centrifuged repeatedly without agglutinating, indicating their good stability (Supplementary Figure S3B). Supplementary Figure S4 illustrates the Raman intensities of the dual DTBN-modified SiO_2_/Au NPs detected 30 times in five separate batches. The difference in SERS intensities was 2.76%, indicating good repeatability.

### Optimization of SERS-Based LFIA Strips

Dual DTNB-modified SiO_2_/Au NPs are very suitable as high-performance SERS probe for LFIA-based bacterial detection due to their excellent SERS performance, superior dispersity, and good stability and repeatability. In this study, *E. coli* O157:H7 was used to evaluate the performance of the SiO_2_/Au-based SERS-LFIA strips. As shown in Scheme 1B, an *E. coli* O157:H7 detection antibody was conjugated with functionalized carboxyl groups of SiO_2_/Au NPs via carbodiimide chemistry (Wang et al., 2015). The antibody concentration was saturated to ensure effectiveness and stability. The unreacted carboxyl sites of the SiO_2_/Au NPs were blocked by saturated BSA to decrease the non-specific adsorption of immune-SiO_2_/Au NPs on the NC membrane. As shown in Supplementary Figure S5A, the zeta potential of the SiO_2_/Au NPs decreased from −21.5 to −25.5 mV after antibody modification. Supplementary Figure S5B displays the TEM image of *E. coli* O157:H7 combined with the antibody-labeled SiO_2_/Au NPs. These results proved the successful conjugation of the anti-*E. coli* O157:H7 antibody with the SiO_2_/Au NPs.

Herein, the SiO_2_/Au NPs acted as a multifunctional material for visual and SERS detection of target bacteria on the LFIA strips. The usage conditions (such as type of NC membrane, running buffer ingredient, and antibody concentration on the test line) of the LFIA strips must be optimized to achieve their best effect on bacterial detection. Two large-aperture NC membranes (CN 95 and CN 140, with pore sizes of 15 and 8 μm, respectively) were tested because the size of the SiO_2_/Au-bacteria complexes are generally larger than 1 μm. Supplementary Figure S6 shows that the these complexes could pass through the wide channels of the CN 95 membrane, whereas many SiO_2_/Au agglomerates were blocked at the junction of the CN 140 membrane and conjunction pad. The accuracy and repeatability of the proposed LFIA strip was seriously influenced by this blocking phenomenon. Therefore, the CN 95 membrane was chosen as the ideal NC membrane of the SiO_2_/Au-based SERS-LFIA strip. The next optimizing experiment was the running buffer, which could substantially affect the immunoreaction of the test lines and the flow rate of the SiO_2_/Au NPs. PBS and 0.5% PBST (PBS containing 0.5% Tween-20, v/v) were considered because our previous work indicated that PBS solution has no influence on the activity of antibody-labeled nanotags (Qie et al., 2019; Wang et al., 2019c). The moderate concentration of Tween-20 ensured the smooth surface of the SiO_2_/Au-bacterial complexes, enabling easy delivery along the strips. However, the non-specific binding at the test line increased when the strips detected the running buffer without bacteria, as shown in Supplementary Figure S7A. FBS was added into the running buffer to decrease the non-specific combination on the test line. The 0.5% PBST buffer containing 20% FBS (v/v) suppressed the non-specific binding more effectively than that containing 10% FBS (v/v), which did not affect the specific binding of the test line. Therefore, 0.5% PBST containing 20% FBS (v/v) was chosen as the running buffer. The sensitivity of the LFIA strips was also affected by the antibody concentration of the test lines. Supplementary Figure S7B indicates that the test line with 0.4 mg/mL capture antibody had the highest signal-to-noise ratio of the SERS signal. In this study, all the optimal conditions were utilized to achieve superior performance of the SiO_2_/Au-based SERS-LFIA strips for pathogen detection.

### Quantitative Analysis of *E. coli* O157:H7 by SiO_2_/Au-Based SERS-LFIA Strip

**FIGURE 3:**
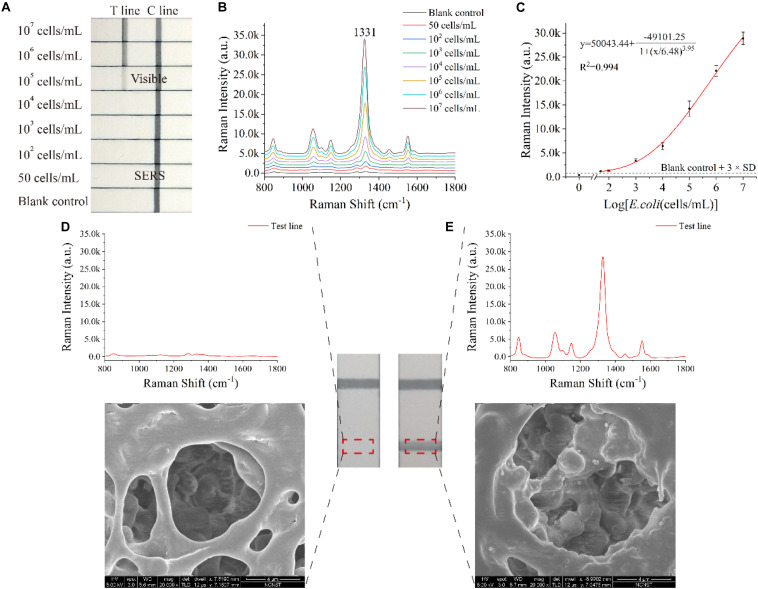
Analytical performance of the dual DTNB-modified SiO_2_/Au-based SERS-LFIA strips. Photographs **(A)**, SERS spectra of the test lines **(B)**, and corresponding calibration curve **(C)** of the LIFA strips for *E. coli* O157:H7 detection. SERS signals, SEM images, and photographs of the test lines of the blank control **(D)** and the positive control **(E)**.

Under optimal conditions (such as NC membrane, running buffer and antibody concentration of test line), *E. coli* O157:H7 sample concentrations ranging from 50 cells/mL to 10^7^ cells/mL were analyzed using the SiO_2_/Au-based SERS-LFIA strip. Figure 3A displays the photo of the SiO_2_/Au-based SERS-LFIA strip testing for *E. coli* O157:H7 detection under different concentrations. When the bacterial concentration was high, the dark color of the SiO_2_/Au NPs immobilizing on the test lines could be clearly observed. This color gradually faded away as the *E. coli* concentration decreased. The visual sensitivity of the dark test lines was 10^5^ cells/mL, and the color was barely visible when the *E. coli* concentration was lower than 10^5^ cells/mL. Thus, quantitative detection of *E. coli* O157:H7 was difficult to conduct with high sensitivity due to the colorimetric change in the test line. However, highly sensitive analysis of *E. coli* O157:H7 was feasible when the SERS signals of these test lines were measured and analyzed using the proposed SiO_2_/Au-based SERS-LIFA strips. Figure 3B illustrates the Raman spectra of the test line with different *E. coli* O157:H7 concentrations ranging from 50 cells/mL to 10^7^ cells/mL in running buffer. The SERS signal intensity declined with the decrease in *E. coli* O157:H7 concentrations. The main peak at 1,331 cm^–1^ was still distinct when the *E. coli* O157:H7 concentrations decreased to 50 cells/mL compared with the blank control. Under SERS detection mode, the Raman intensity of the test lines was measured to quantitatively detect *E. coli* O157:H7. As displayed in Figure 3C, the calibration curves of the SiO_2_/Au-based SERS-LFIA strips were constructed using the Raman intensity from their test lines and the *E. coli* O157:H7 concentrations. Error bars showed the standard deviation (SD) of five detections. The LOD of *E. coli* O157:H7 analyzed using the LFIA strips was 50 cells/mL based on the IUPAC formula (LOD = y_blank_ + 3 × SD_blank_, where y_blank_ and SD_blank_ are the average Raman intensity and the SD of the blank control, respectively) (Hu et al., 2017; Putnin et al., 2019). The SiO_2_/Au-based SERS-LFIA strips were characterized by SEM to prove that the SERS signal came from the SERS tags fixed on the test line. Figure 3D illustrates the SEM image of the test line of the negative sample. The color and SERS signal remained unchanged, and SERS tags could not be observed on the test line. Figure 3E displays the SEM image of the test line of the positive sample. The color of the test line became dark, and the Raman intensity was enhanced as the SERS tags were gradually immobilized on the test area. These results directly proved that SiO_2_/Au-bacterial complexes were immobilized on the test zone by forming sandwich-like immunocomplexes.

An ordinary SERS-based LFIA strip was prepared by utilizing the same paired *E. coli* O157:H7 antibody and replacing the SiO_2_/Au NPs with common colloidal Au NPs (50 nm) to prove the superior performance of the proposed SiO_2_/Au-based SERS-LFIA strips. The preparation of colloidal Au SERS tags-based LFIA strips and the detection method of *E. coli* O157:H7 are described in Supplementary Material. As shown in Supplementary Figure S8, the LOD of the colloidal Au SERS tags-based LFIA strip was 10^5^ cells/mL on vision, while that of *E. coli* O157:H7 under SERS detection mode was 10^4^ cells/mL. Thus, the SERS sensitivity of SiO_2_/Au-based SERS-LFIA strip was 2,000 and 200 times higher than the visual and SERS sensitivity of colloidal Au SERS tags-based LFIA strip, respectively. Moreover, the SiO_2_/Au based SERS-LFIA strip enabled fast bacterial detection within 15 min, which included a 12 min chromatographic process and 3 min SERS analysis time.

**FIGURE 4:**
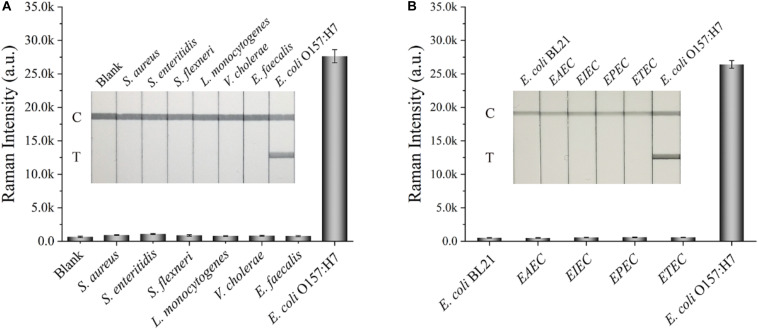
Specificity of the dual DTNB-modified SiO_2_/Au-based SERS-LFIA strip. Images of the strips and corresponding Raman intensities of the test lines for several enteropathogenic bacteria **(A)** and other non-O157 serotype *E. coli* **(B)**. The concentration of these bacteria was approximately 10^7^ cells/mL.

Other indexes, such as specificity, reproducibility, and stability, were also considered to further assess the performance of the SiO_2_/Au-based SERS-LFIA strips. Several enteropathogenic bacteria, such as *S. aureus, S. enteritidis, S. flexneri, L. monocytogenes, V. cholera*, and *E. faecalis*, were used as interferents to test the specificity of the proposed assay. All the bacterial samples were detected by the developed strip and prepared at a concentration of approximately 10^7^ cells/mL. Figure 4A shows that the Raman intensity of all these non-target bacteria was close to the intensity of the blank control, but the signal of *E. coli* O157:H7 group was obvious on the test line. Moreover, other non-O157:H7 serotype of *E. coli* such as *E. coli* BL21, *EAEC, EIEC, EPEC*, and *ETEC* were detected to further verify the specificity of the SiO_2_/Au-based SERS-LFIA strips, as shown in Figure 4B. Results indicate excellent specificity of the SiO_2_/Au-based SERS-LFIA strips for target bacterial detection. The reproducibility of these strips was studied by testing tap-water samples, which were spiked with different *E. coli* O157:H7 concentrations. Five independent experiments were performed to estimate 10^7^ and 10^4^ cells/mL of *E. coli* O157:H7 samples. Supplementary Figure S9 shows that the relative SD values of the SERS intensity were 5.03% and 6.92%, respectively, indicating high reliability and reproducibility of the prepared SERS-LFIA strips.

### Detection of *E. coli* O157:H7 in Tap Water and Biological Samples

**FIGURE 5:**
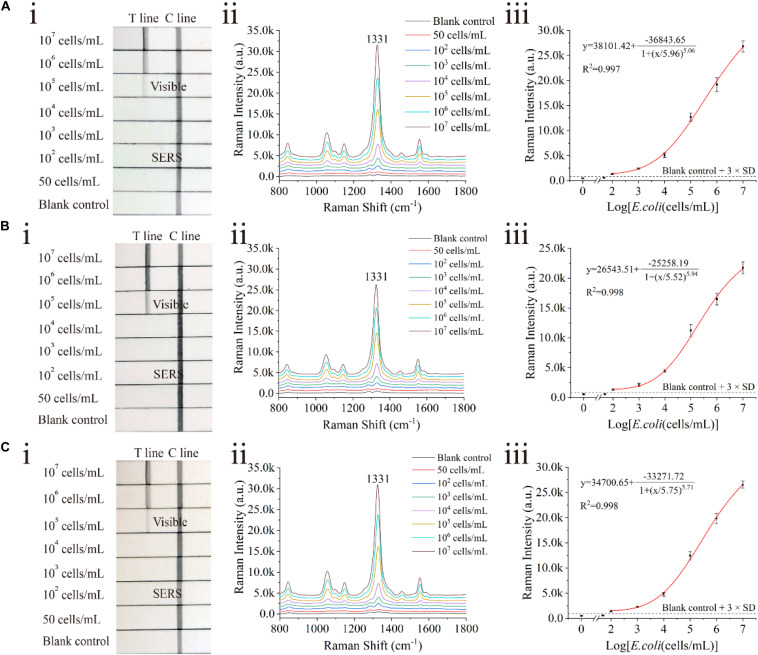
Analytical performance of dual DTNB-modified SiO_2_/Au-based SERS-LFIA strip in biological samples. Photographs (i), Raman intensities of the test lines (ii), and corresponding calibration curves (iii) of the LFIA strips for *E. coli* O157:H7 detection in tap water **(A)**, milk **(B)**, and human urine **(C)**.

The proposed assay detected *E. coli* O157:H7 in tap water and biological samples, including milk and human urine, to evaluate the stability and practicability of the proposed LFIA strips. *E. coli* O157:H7 was spiked into these samples with different concentrations ranging from 50 cells/mL to 10^7^ cells/mL. In the detection process, 80 μL of the sample solution was mixed with 20 μL of the diluent (FBS containing 2.5% Tween-20, v/v) and pipetted onto the sample pad of the SiO_2_/Au-based SERS-LFIA strip. Using the proposed LFIA system, *E. coli* O157:H7 was accurately detected in the biological samples. As shown in Figure 5, the Raman signals of these positive samples were distinguished but slightly weaker than those in the running buffer. Moreover, no Raman signals could be achieved in the negative groups. The LODs of *E. coli* O157:H7 in tap water (Figure 5A), milk (Figure 5B), and human urine (Figure 5C) were as low as 100 cells/mL. Such low values met the clinic test requirement (Ho et al., 2019). To further verify the practicality of the SiO_2_/Au-based SERS-LFIA strips, *E. coli* O157:H7 was detected in other food samples, such as lettuce extract and beef. As shown in Supplementary Figure S10, the sensitivity of the SiO_2_/Au-based SERS-LFIA strip was not influenced by the food samples. It was the high stability of the SiO_2_/Au particles that ensured the superior anti-interference ability of this method for biological samples.

Table 1 shows the comparison between results from the current study and those from other reported LFIAs for bacterial detection. The SiO_2_/Au based SERS-LFIA strip considerably improved the detection sensitivity of the target bacteria in comparison with other previously reported LFIA system (10^2^–10^5^ cells/mL), especially the commercial colloidal Au strip (10^5^–10^7^ cfu/mL) (Gast et al., 2003; Kong et al., 2017; Hu et al., 2018; Shin et al., 2018; Wang et al., 2018a; Huang et al., 2019; Zhang et al., 2020). In addition, the SiO_2_/Au-LFIA strip could be successfully used for quantitative detection of *E. coli* O157:H7 in biological samples, making the POCT promising for sensitive testing of pathogenic bacteria. A notable detail that the great reproducibility and specificity of this strip were too dependent on the specific *E. coli* antibody utilized on the SiO_2_/Au labels and test lines. In theory, sensitive detection could also be conducted on other pathogenic bacteria via the SiO_2_/Au-LFIA strip by using their corresponding specific antibodies.

**TABLE 1:** Comparison of the LOD for bacteria detections by different LFIAs.

Type of label	Bacteria	LOD (cells/mL)	Assay time	References
Au NPs	*E. coli* O157:H7	1.87 × 10^4^	19 min	Shin et al., 2018
	*S. typhimurium*	1.47 × 10^4^	19 min	
Au@Ag NPs	*E. coli* O157:H7	5 × 10^4^	15 min	Liu et al., 2019
Nitrogen-rich carbon NPs	*S. enteritidis*	10^2^	20 min	Wang Z. et al., 2019
Au NPs	*Bacillus cereus*	10^4^	20 min	Kong et al., 2017
Fluorescent-magnetic NPs	*S. typhimurium*	3.5 × 10^3^	35 min	Hu et al., 2018
SiO_2_/Au NPs	*E. coli* O157:H7	50	15 min	This work

## Conclusion

In conclusion, a new type of dual DTNB molecule-modified SiO_2_/Au NP was developed using an efficient PEI-mediated seed growth method. PEI was utilized to form an interlayer with positive charge into the nanostructure, which effectively absorbed a mass of 3–5 nm Au/DTNB NPs as seeds for inner DTNB loading and Au shell reduction. The obtained SiO_2_/Au nanocomposites possessed excellent stability and monodispersity and superior SERS properties. They were applied in the LFIA strips as high-performance SERS tags for bacterial detection in high sensitivity. The feasibility of the SiO_2_/Au-based SERS-LFIA system was verified by detecting *E. coli* O157:H7. The SiO_2_/Au-based SERS-LFIA strips were proven to be an effective tool for the specific detection of *E. coli* in biological samples and the SERS quantitative analysis of target bacteria on the test line of the strip under optimal experimental conditions. The LOD of *E. coli* was 50 cells/mL, indicating that these strips were 200 and 2,000 times more sensitive than the SERS and visual LOD of the colloidal Au SERS tags-based LFIA strips, respectively. Moreover, the high stability of the SiO_2_/Au particles ensured the good practicability of our proposed strip for biological samples. To our knowledge, this study was the first to utilize SiO_2_/Au-based SERS-LFIA strip for bacterial detection. Considering other advantages, such as shortened detection time, easy operation, great reproducibility, and low cost (<$1 per test), the proposed assay has a good potential for rapid and sensitive detection of pathogenic bacteria in biological samples.

## Data Availability Statement

The original contributions presented in the study are included in the article/Supplementary Material, further inquiries can be directed to the corresponding authors.

## Author Contributions

BG, SW, and CW designed and managed the project. LS, LX, RX, and ZZ performed all the experiments. LS and LX did the analysis of SERS immunoassay results. BG, CW, and LS wrote the manuscript. All authors reviewed the manuscript.

## Conflict of Interest

The authors declare that the research was conducted in the absence of any commercial or financial relationships that could be construed as a potential conflict of interest.

## Footnotes

1 https://www.reagent.com.cn

2 https://www.sigmaaldrich.com

3 https://www.thermofisher.com

4 http://www.xinxinbio.com

5 http://www.joey-bio.com

6 https://www.sartorius.com.cn

7 http://www.bnbio.com
