# Supplementary material for: Rapid, Quantitative, High-Sensitive Detection of Escherichia coli O157:H7 by Gold-Shell Silica-Core Nanospheres-Based Surface-Enhanced Raman Scattering Lateral Flow Immunoassay
Source: Front Microbiol. 2020 Nov 6;11:596005. doi: 10.3389/fmicb.2020.596005 (PMC7677456; doi:10.3389/fmicb.2020.596005)
Supplement: Supplementary file 1 [file Data_Sheet_1.docx]

Supporting Information

**Rapid, quantitative, high-sensitive detection of *Escherichia coli* O157:H7 by gold-shell silica-core nanospheres-based Surface-enhanced Raman scattering lateral flow immunoassay**

Luoluo Shi^a,b,c,1^, Ling Xu^a,c,1^, Rui Xiao^b^, Zihui Zhou^d^, Chongwen Wang^a,b,d,^*^*^*, Shengqi Wang^b,c,^*^*^*, Bing Gu^a,c,^*^*^*

*^a^ Medical Technology Institute of Xuzhou Medical University, Xuzhou 221004, PR China.*

*^b^ Beijing Institute of Radiation Medicine, Beijing 100850, PR China.*

*^c^ Department of Laboratory Medicine, Affiliated Hospital of Xuzhou Medical University, Xuzhou 221004, PR China.*

*^d^ College of Life Sciences, Anhui Agricultural University, Hefei 230036, PR China.*

**1. Preparation of colloidal Au SERS-tags.**

Firstly, 20 μL of 10 mM DTNB ethanol solution was mixed to 10 mL of Au NPs solution. The mixture was under vigorous stirring at room temperature for 4 h. Then, 2 mL of colloidal Au/DTNB NPs were centrifuged to eliminate the superfluous DTNB. Afterward, the pellet was resuspended in 1 mL of MES buffer (2 mM, pH 5.5). Freshly prepared EDC (10 mM, 10 μL) and NHS (10 mM, 20 μL) were added into the mixture and vigorously shake for 15 min. Subsequently, the mixture was centrifuged and resuspended in 1 mL of PB (2 mM). *E. coli* O157:H7 detection antibody (20 μg) was added into the mixture and incubated for 2 h. Then, 100 μL of 10% BSA (w/v) was added to block the unreacted carboxyl sites of SERS labels for an additional 1 h at 37 °C. The functionalized SERS labels were pelleted by centrifugation and resuspended in 200 μL of a solution containing Tris–HCl (50 mM, pH 8.0), 0.5% Tween-20 (v/v), 1% BSA (w/v), 10% sucrose (w/v), and 0.1% PVP (w/v). The SERS tags were evenly pipetted onto the glass fiber paper and dried at 37 °C to form conjugate pads.

**2. Preparation of colloidal Au SERS tags-based LFIA strips**

The colloidal Au SERS tags-based LFIA strips was composed of four sections: sample pad, absorbent pad, conjugate pad, and NC membrane. *E. coli* O157:H7 capture antibody (0.4 mg/mL) and goat anti-mouse IgG antibody (1 mg/mL) were drawn onto the NC membrane separately and dried at 37 °C for 1 h. Then, the four sections were assembled onto a PVC bottom plate in sequence. The integrated membrane was cut into 3 mm-wide strips with a paper cutter and stored in a dry container until use.

**3.** ***E. coli* O157:H7 detection using the colloidal Au SERS tags-based LFIA strips**

Briefly, *E. coli* O157:H7 was diluted in running buffer (2mM PB containing 20% FBS and 0.5% Tween-20, v/v), with concentrations ranging from 10^2^ cells/mL to 10^7^ cells/mL, as positive samples. The running buffer without *E. coli* O157:H7 was used as the blank control. Then, 100 μL of sample solution were pipetted onto the sample pad and moved toward the absorbent pad under the capillary effect. 12 minutes later, detecting the SERS signals of test lines with a portable Raman spectrometer. The integration time and laser power were 10 s and 10 mW, respectively.


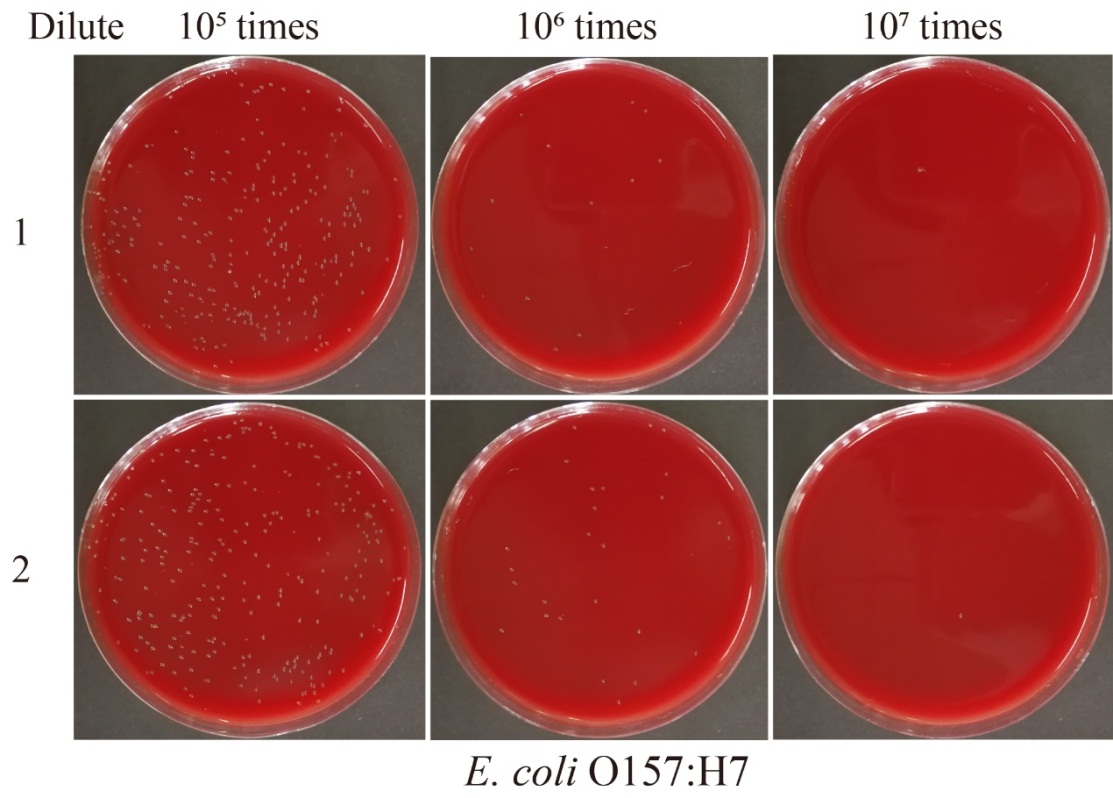


**Fig. S1.** Photographs of *E. coli* O157:H7 colony growth on the blood agar plates. The original *E. coli* concentration was diluted 10^5^, 10^6^ and 10^7^ times respectively.


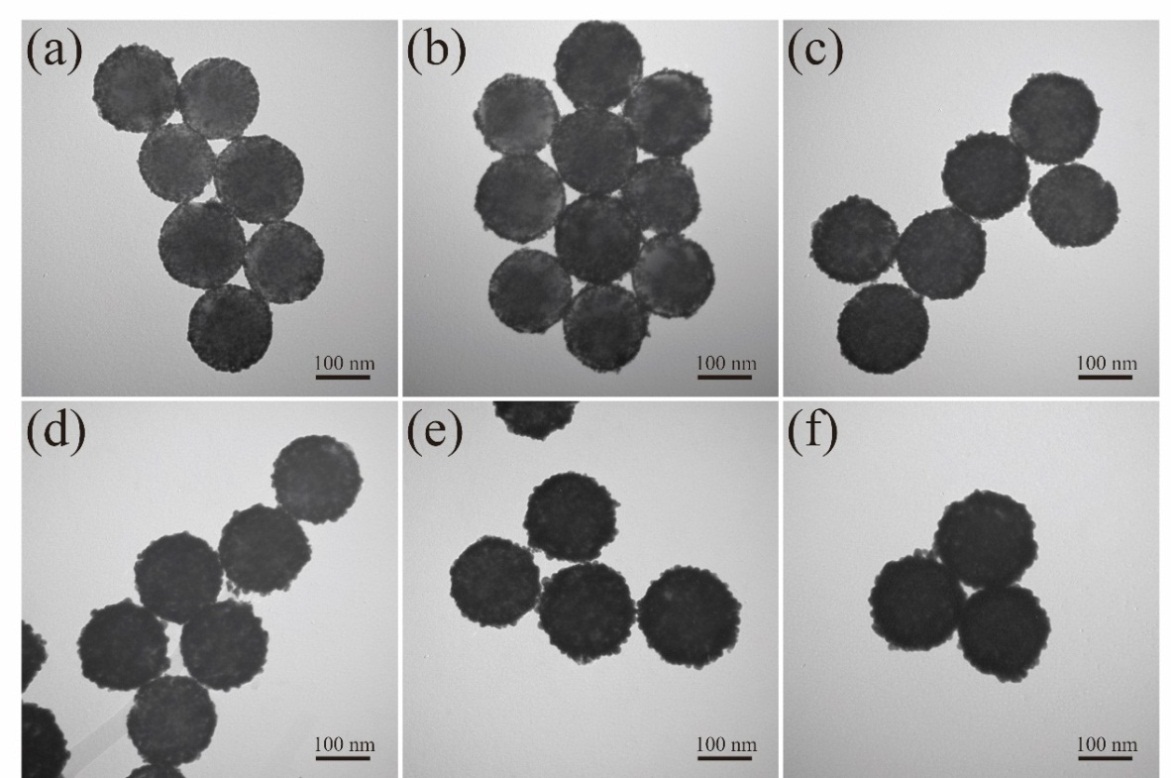


**Fig. S2.** TEM images of SiO_2_/Au NPs synthesized with different HAuCl_4_ concentrations: (a-f) 0.01, 0.02, 0.03, 0.04, 0.05, 0.06 mM HAuCl_4_.


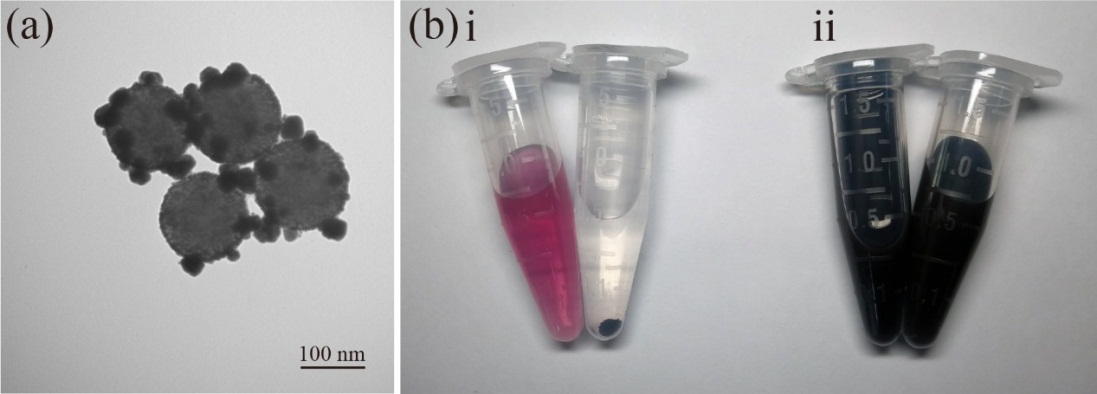


**Fig. S3.** (a) TEM image of SiO_2_/Au NPs synthesized with the DTNB concentrations over 50 μM. (b) image of colloidal Au NPs (i) and SiO_2_/Au NPs (ii) in high ion solutions, the colloidal Au NPs was soon agglutinated while the SiO_2_/Au NPs was still suspended.





**Fig. S4.** The Raman intensity of dual DTNB-modified SiO_2_/Au NPs, which were detected in 30 times in 5 independent batches.


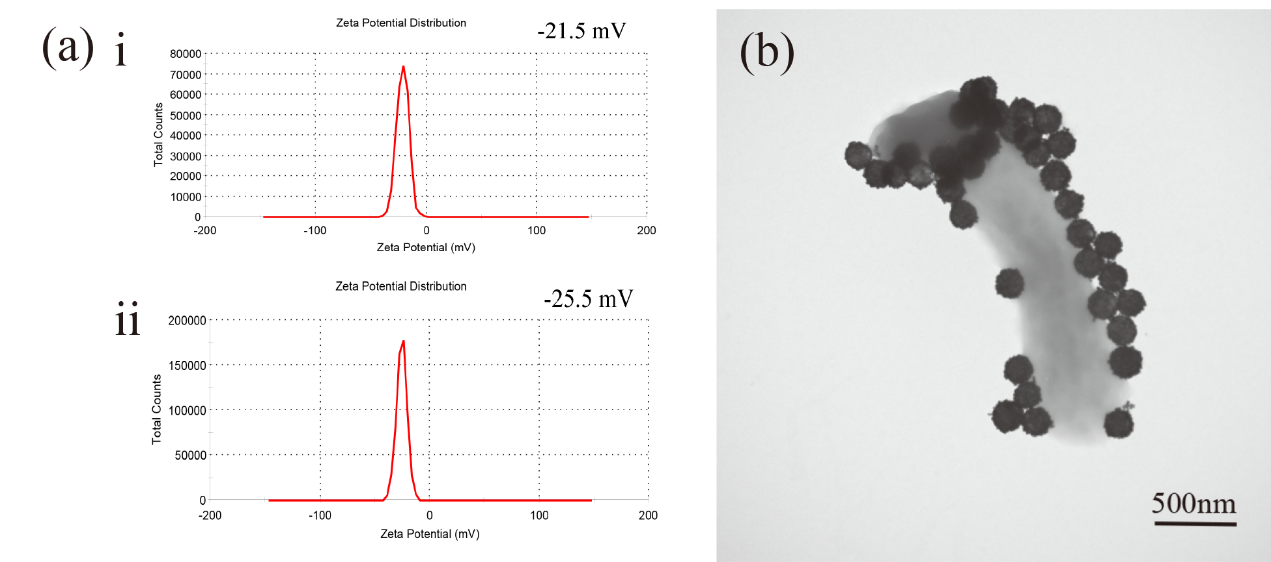


**Fig. S5.** (a) The zeta potentials of SiO_2_/Au NPs (i) and SERS tags (ii). (b) The TEM image of SERS tags combining with the *E. coli* O157:H7.


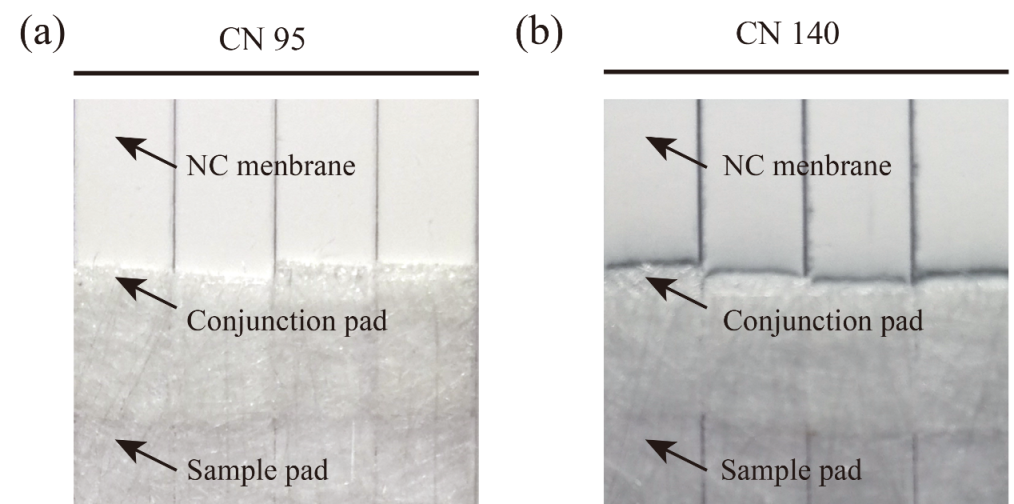


**Fig. S6.** Effects of CN95 (a) and CN140 (b) membranes on the LFIA strips. The tags and bacteria complexes were easily blocked at the junction of conjugate pad and CN140 membrane.


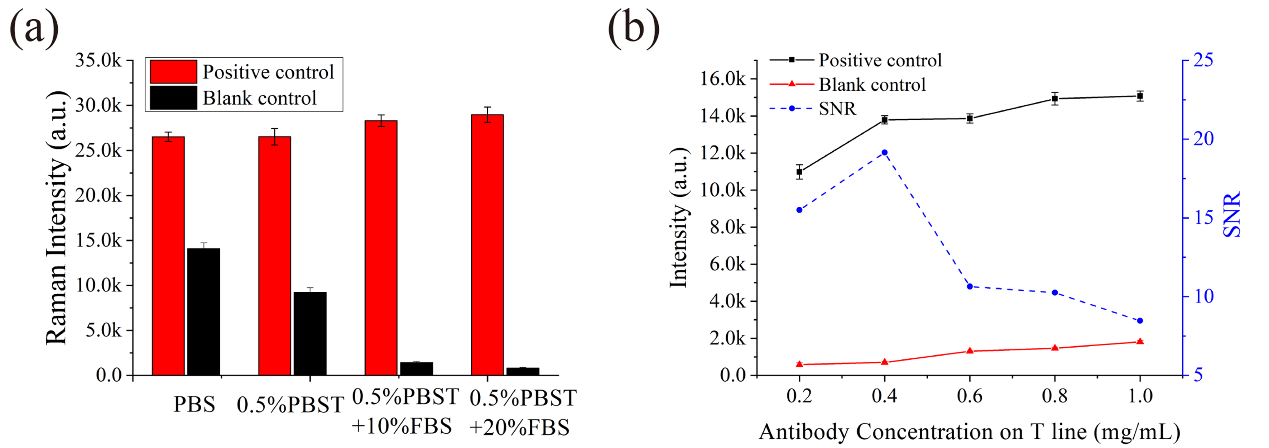


**Fig. S7.** Optimization of the running buffer (a) and the antibody concentration of the test lines (b).


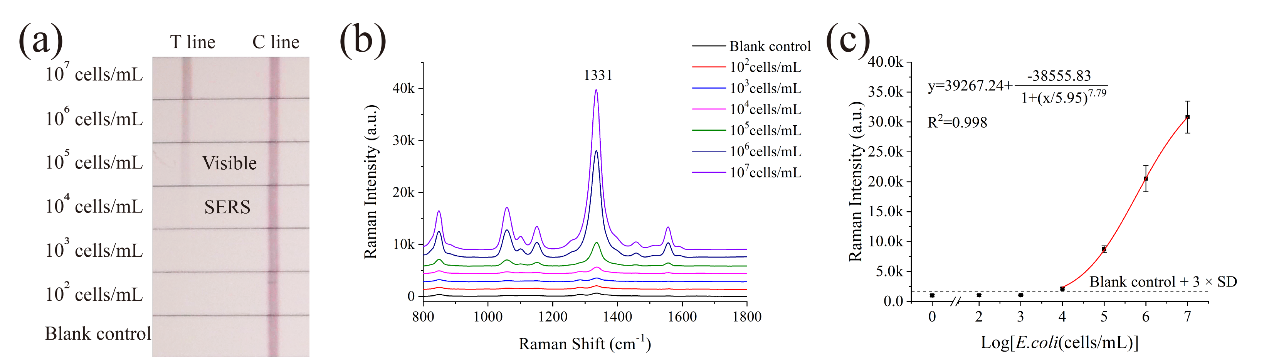


**Fig. S8.** Analytical performance of the colloidal Au-based LFIA strips. Photograph (a). SERS spectra of the test lines (b), and corresponding calibration curve (c) of the LIFA strips for *E. coli* O157:H7 detection.


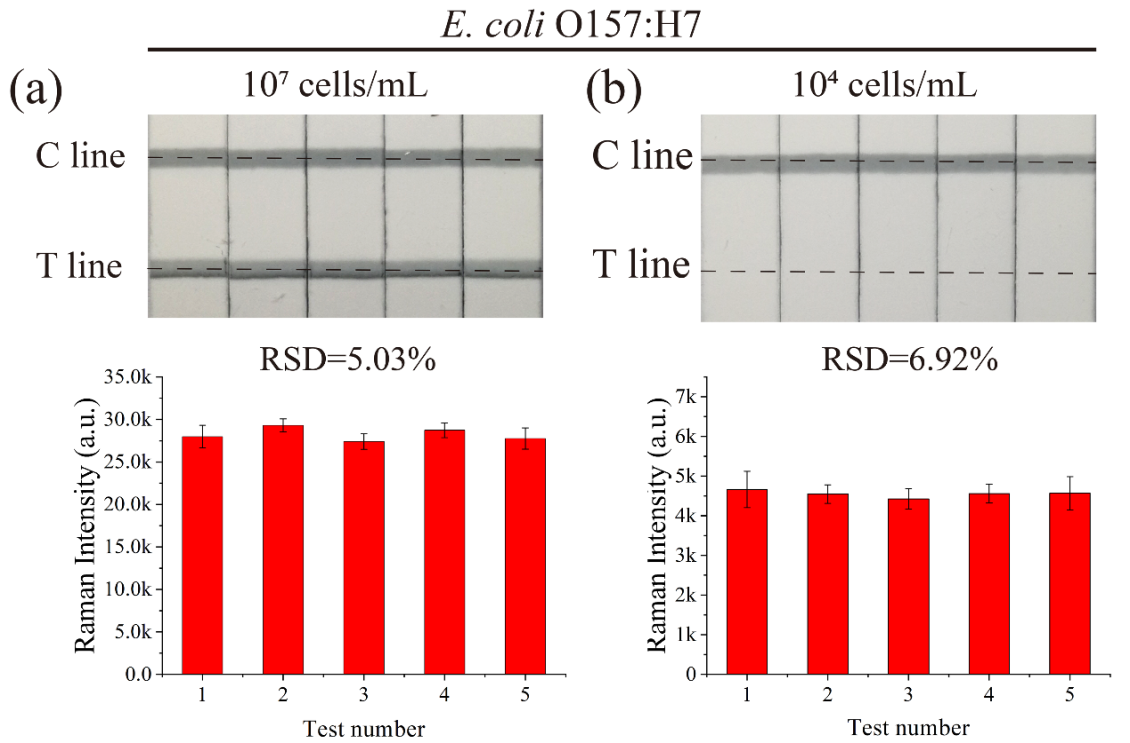


**Fig. S9.** Assay reproducibility of five tests of *E. coli* O157:H7 at a concentration of 10^7^ cells/mL (a) and 10^4^ cells/mL (b) in tap-water. The error bars represent the standard deviations from five separate experiments.


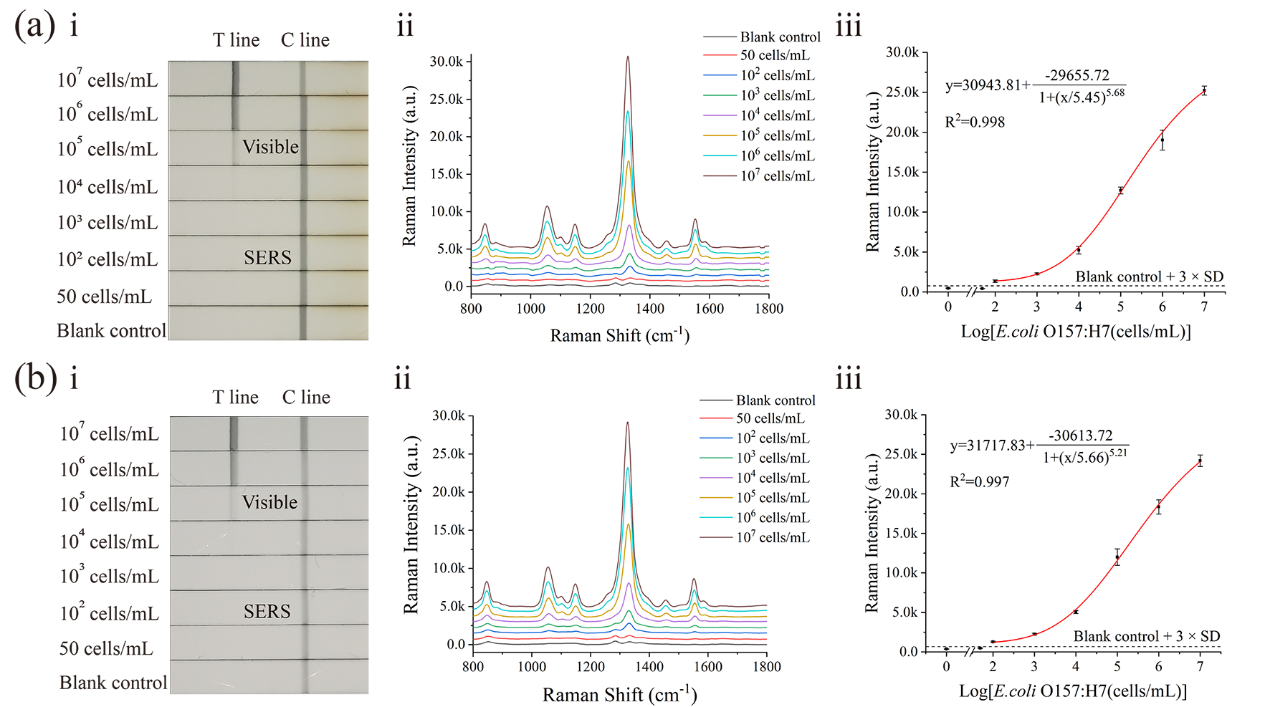


**Fig. S10.** Analytical performance of dual DTNB-modified SiO_2_/Au-based SERS-LFIA strip in food samples. Photographs (i), SERS signals of the test lines (ii,) and corresponding calibration curves (iii) of the LFIA strips for *E. coli* O157:H7 detection in lettuce extract (a) and beef (b) samples.
